# Assessing the efficiency of catch-up campaigns for the introduction of pneumococcal conjugate vaccine: a modelling study based on data from PCV10 introduction in Kilifi, Kenya

**DOI:** 10.1186/s12916-017-0882-9

**Published:** 2017-06-07

**Authors:** Stefan Flasche, John Ojal, Olivier Le Polain de Waroux, Mark Otiende, Katherine L. O’Brien, Moses Kiti, D. James Nokes, W John Edmunds, J. Anthony G. Scott

**Affiliations:** 1 Department of Infectious Disease Epidemiology, London School of Hygiene and Tropical Medicine, Keppel Street, WC1E 7HT London, UK; 2 Kenya Medical Research Institute (KEMRI)-Wellcome Trust Research Programme, Centre for Geographic Medicine Research-Coast, Kilifi, Kenya; 3 International Vaccine Access Center, Johns Hopkins Bloomberg School of Public Health, Baltimore, MD USA; 4 University of Warwick and WIDER, Coventry, UK

**Keywords:** Pneumococcus, Catch-up, Vaccination, PCV, Dose efficiency, Impact

## Abstract

**Background:**

The World Health Organisation recommends the use of catch-up campaigns as part of the introduction of pneumococcal conjugate vaccines (PCVs) to accelerate herd protection and hence PCV impact. The value of a catch-up campaign is a trade-off between the costs of vaccinating additional age groups and the benefit of additional direct and indirect protection. There is a paucity of observational data, particularly from low- and middle-income countries, to quantify the optimal breadth of such catch-up campaigns.

**Methods:**

In Kilifi, Kenya, PCV10 was introduced in 2011 using the three-dose Expanded Programme on Immunisation infant schedule and a catch-up campaign in children <5 years old. We fitted a transmission dynamic model to detailed local data, including nasopharyngeal carriage and invasive pneumococcal disease (IPD), to infer the marginal impact of the PCV catch-up campaign over hypothetical routine cohort vaccination in that setting and to estimate the likely impact of alternative campaigns and their dose efficiency.

**Results:**

We estimated that, within 10 years of introduction, the catch-up campaign among children <5 years old prevents an additional 65 (48–84) IPD cases across age groups, compared to PCV cohort introduction alone. Vaccination without any catch-up campaign prevented 155 (121–193) IPD cases and used 1321 (1058–1698) PCV doses per IPD case prevented. In the years after implementation, the PCV programme gradually accrues herd protection, and hence its dose efficiency increases: 10 years after the start of cohort vaccination alone the programme used 910 (732–1184) doses per IPD case averted. We estimated that a two-dose catch-up among children <1 year old uses an additional 910 (732–1184) doses per additional IPD case averted. Furthermore, by extending a single-dose catch-up campaign to children aged 1 to <2 years and subsequently to those aged 2 to <5 years, the campaign uses an additional 412 (296–606) and 543 (403–763) doses per additional IPD case averted. These results were not sensitive to vaccine coverage, serotype competition, the duration of vaccine protection or the relative protection of infants.

**Conclusions:**

We find that catch-up campaigns are a highly dose-efficient way to accelerate population protection against pneumococcal disease.

**Electronic supplementary material:**

The online version of this article (doi:10.1186/s12916-017-0882-9) contains supplementary material, which is available to authorized users.

## Background

With the aid of Gavi, the Vaccine Alliance (Gavi for short), many low-income countries, in particular across Africa, have introduced pneumococcal conjugate vaccines (PCVs) into their infant immunisation programmes. However, there remain Gavi countries, particularly in south Asia and northern Africa and some with large infant populations, who are yet to follow [1]. Country policy makers, along with global stakeholders, have a high interest in achieving optimal health impact from PCVs as quickly as possible; however, approaches for achieving maximum and rapid impact have to be weighed against relative cost. In situations where vaccine supply is constrained, as was the case several years ago for PCVs, issues of efficiency and equity in vaccine use are also a consideration [2]. The World Health Organisation (WHO) recommends that catch-up campaigns can be used as part of the introduction of PCVs to accelerate the build-up of herd protection and hence PCV impact [3]. However, it is unclear if such catch-up campaigns are an efficient way to use PCVs or if the gains from this approach are less than the relative increase in the number of doses required.

The value of a catch-up campaign is assessed by quantifying the trade-off between the costs of vaccinating additional age groups and the benefit of additional direct and indirect protection. However, there are few observational data on the impact of PCV campaigns, particularly from low- and middle-income countries (LMICs), to quantify the optimal approach of catch-up campaigns. One of the few well-studied examples of a PCV introduction catch-up campaign in an LMIC occurred in Kilifi, Kenya. The 10-valent pneumococcal non-typeable *Haemophilus influenzae* protein D-conjugate vaccine (PCV10) was introduced into the Kenyan routine childhood vaccination programme in early January 2011 using the WHO Expanded Programme on Immunisation (EPI) schedule of three infant doses at 6, 10 and 14 weeks. Additionally, in Kilifi County, at the introduction of the cohort programme, a three-dose catch-up campaign was offered to all infants younger than 12 months of age and a two-dose catch-up to children 12–59 months of age.

We fitted a transmission dynamic model of pneumococcal carriage (a precondition for disease and the source of person-to-person community transmission) and disease to detailed pre- and post-PCV introduction data from Kilifi. This allowed us to quantify the marginal impact of the PCV catch-up campaign on carriage and disease in Kilifi over the hypothetical impact of a routine cohort vaccination programme alone in that setting. Using this framework, we aimed to estimate the dose efficiency of alternative catch-up campaigns in relation to PCV cohort introduction alone.

## Methods

### Data

#### Study population and mixing patterns

Kilifi County is a mainly rural area on the Indian Ocean coast of Kenya. The Kilifi Health and Demographic Surveillance System (KHDSS) was established in 2000. Approximately 260,000 people reside in the KHDSS area, and 60% are younger than 20 years of age [4]. Within the KHDSS numerous studies regarding pneumococcus and its health effects have been conducted that informed this work (Table 1). The demographic structure of the model is based on 2009 mid-year population census estimates and assumes no demographic changes with time. To adjust for changes in the population age distribution, we used respective annual mid-year population estimates to calculate the invasive pneumococcal disease (IPD) incidence rates. A cross-sectional prospective diary-based contact survey was conducted in the northern part of the KHDSS area in 2009 [5, 6]. In total, 623 randomly selected participants of all ages produced 568 completed diaries in which they reported their contacts during 24 hours and reported 27,395 physical (i.e. skin-to-skin) contacts with 10,042 unique individuals. This information was used as a proxy for transmission of pneumococcal carriage [7, 8]. Standard methods were used to calculate the WAIFW (Who Acquires Infection From Whom) mixing matrix for age groups <1 year, 1–5 years, 6–15 years, 16–19 years, 20–49 years and older than 50 years for the KHDSS [5, 8–10].

**Table 1:** Overview of model parameters

Model parameters		Contribution	No. of parameters	Prior				Posterior	
				Distribution	Mean	SD	Source	Median	CR
Study population	Age group size	Fixed	6 age groups	–	–	–	KHDSS [4]	–	–
	Contact patterns	Fixed	6 × 6 age groups	–	–	–	KHDSS [5]	–	–
	Carriage prevalence	Outcome	2 types × 6 age groups	–	–	–	KHDSS [11]	–	–
	IPD	Outcome	2 types × 6 age groups	–	–	–	KHDSS [12]	–	–
	Observed vaccine coverage	Fixed	27,040 (weekly age and time)	–	–	–	KHDSS [4]	–	–
Transmission dynamics	Clearance rates	Fixed	2 types × 4 age groups				KHDSS [15]		
	VE carriage (toddlers)	Fitted - prior	1	Normal	0.36	0.15	KHDSS [11]	0.56	0.41–0.72
	VE IPD (infants)	Fitted - prior	1	Normal	0.80	0.1	[22]	0.86	0.67–0.99
	Duration of protection (toddlers)	Fitted - prior	1	Normal	6 years	3	[23, 24]	5.50	2.05–11.01
	Relative level of infant protection	Fitted - prior	1	Normal	1	0.1	Assumption	0.97	0.78–1.17
	Competition parameter	Fitted - prior	1	Log-normal	0.15	0.15	[17, 18]	0.19	0.07–0.44
	Susceptibility to infection	Fitted - no prior	2 types × 4 age groups						
	Invasiveness	Fitted - no prior	2 types × 4 age groups						

*CR* Credible range, *VE* Vaccine efficacy

#### Pneumococcal carriage and IPD

The model was fitted to vaccine type (VT) and non-vaccine type (NVT) carriage prevalence and IPD incidence between 2009 and 2015 and between 2008 and 2015, respectively. During that period annual cross-sectional carriage surveys were conducted in the KHDSS area [11]. In each study a nasopharyngeal swab was collected from more than 500 randomly selected individuals of all ages. Surveillance with passive case finding for IPD was introduced at Kilifi County Hospital in 1998 for children and in 2007 for adults. Among the residents of the KHDSS area, 30–70 cases of IPD have been reported annually [12]. Much of that variation is due to changes in disease caused by serotype 1, which has been reported previously to be unstable in various settings [13, 14]. As the mechanisms behind serotype 1 epidemic behaviour are poorly understood, we used multiple pre-vaccination years to include this variance into our baseline for the predictions.

#### Duration of carriage

We used average age-specific pneumococcal colonisation clearance rates estimated from a longitudinal carriage survey in the KHDSS area [15] and reported for the age groups <22 months, 22–40 months and 41–59 months. Based on other studies [16], we assumed that clearance rates in individuals older than 5 years were 60% higher than in children of age 2–4 years.

#### Serotype competition

The competition parameter, which determines the proportion by which the likelihood of acquisition is reduced by heterologous carriage, based on local data was only estimated as serotype specific [15] rather than for pooled VT and NVT groups. Thus, we used a log-normal prior distribution with a median of 0.11 based on estimates from other settings [17–19].

#### Vaccine coverage

As part of the KHDSS, electronic individual-based records of the delivery of vaccines are routinely collected at vaccine clinics [20]. We calculated weekly estimates of PCV coverage for the 2 years after PCV introduction; each was stratified by weekly age cohorts from newborns up to 5 years of age (Additional file 1: Figure S2). Two such coverage estimates were calculated: vaccine coverage of at least two doses of PCV administered before the age of 1 year, which was deemed ’infant protection’, and vaccine coverage of at least one dose of PCV administered after the age of 1 year, deemed ’toddler protection’. The choice of at least two doses for infants and at least one dose for toddlers was made on the basis of observed coverage rates. For calculation of the number of doses used, we assumed that vaccinated infants within the routine programme received three doses, infants vaccinated as part of the catch-up received two doses and toddlers received one dose. Data for vaccination rates were available only through late 2012; we extrapolated those rates forward in time by assuming the coverage rates as of later 2012 to continue for the rest of the study period.

#### Vaccine efficacy

The efficacy against VT nasopharyngeal carriage of a single dose of PCV10 administered to children 12–59 months old has been estimated in a randomised controlled trial in Kenya at 36% (95% confidence interval: –1 to 60) [21]. We further assumed that vaccine efficacy of PCV10 against VT IPD of a complete primary series was 80% based on a meta-analysis for PCVs for infants elsewhere [22]. These two estimates of vaccine efficacy against VT carriage and VT IPD were used as priors in the fitting process. Those who were vaccinated in infancy, i.e. before 1 year of age, may have different vaccine efficacy against acquisition of colonisation, different vaccine efficacy against progression to invasive disease and different duration of protection than vaccinated toddlers. We allowed for the model to estimate these three parameters for infants as a common proportion of respective parameters for toddlers, under the null hypothesis that no difference exists.

#### Duration of vaccine protection

As estimates of the duration of protection from PCV were not available from studies within the KHDSS, we used estimates derived from external studies. Hence our prior on the average duration of protection against carriage and disease is centred around 6 years [16, 23, 24]. Vaccine protection is modelled as leaky protection [25].

### Model

We used a Susceptible, Infected and Infectious, Susceptible type model of the transmission of grouped vaccine and non-vaccine pneumococcal serotypes as described previously [16, 17]. The group of vaccine serotypes consisted of all pneumococcal serotypes targeted by PCV10, i.e. serotypes 1, 4, 5, 6B, 7 F, 9 V, 14, 18C, 19 F and 23 F. Individuals were grouped into compartments by their age (weekly age groups until 5 years of age and yearly age groups thereafter), their infection status (either susceptible, infected with a vaccine serotype, a non-vaccine serotype or both at the same time) and by their vaccination status (unprotected, infant protection, toddler protection). At acquisition of carriage an age-group- and serotype-group-specific proportion of carriers develop disease. These proportions were estimated from the model alongside vaccine efficacy against carriage and IPD, duration of protection, the relative level of infant protection, the competition parameter and the susceptibility to infection (Table 1).

**Fig. 1:**
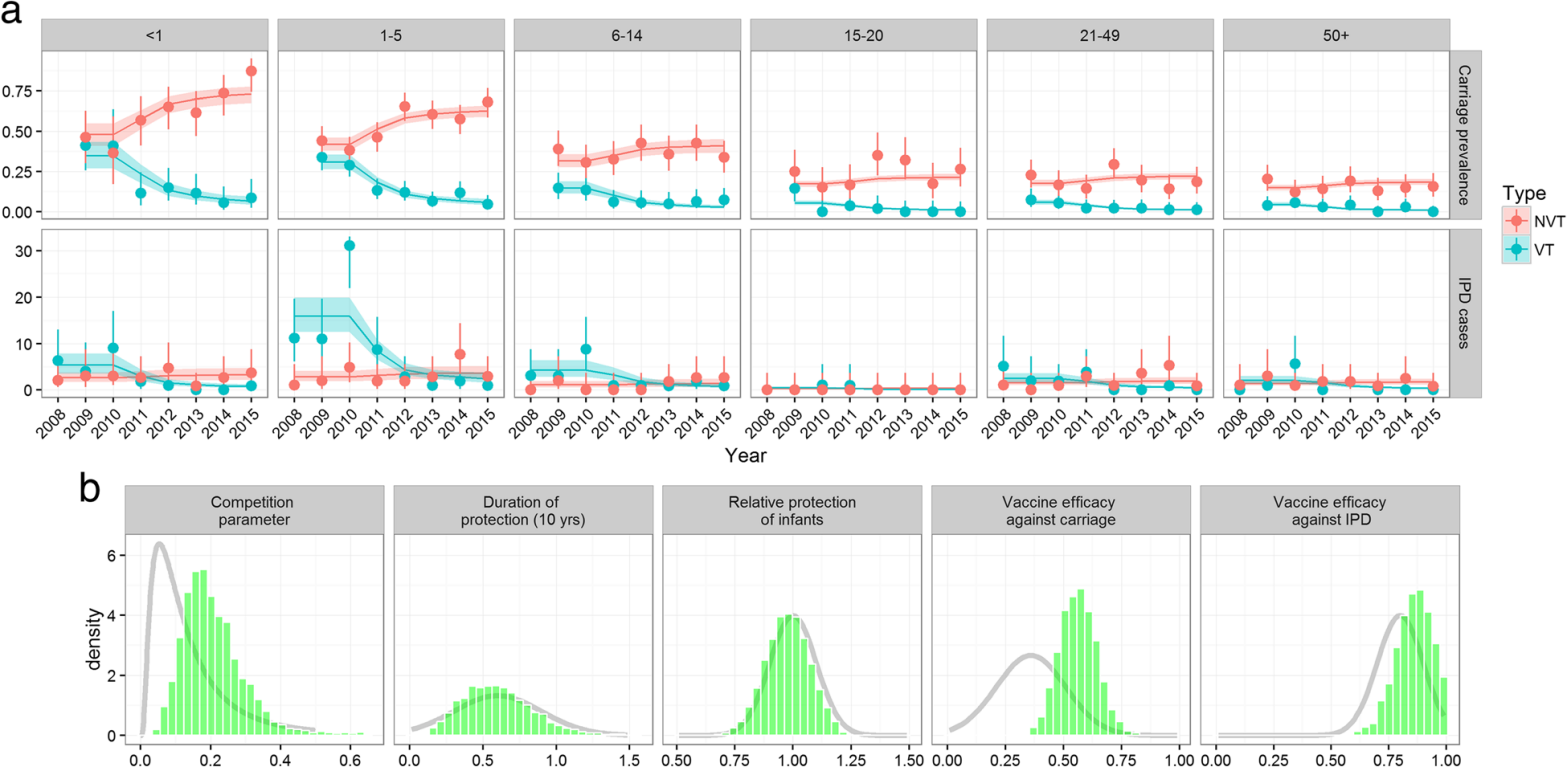
Model fit to carriage prevalence and IPD incidence (**a**) and prior and posterior parameter estimates (**b**). We assumed that serotyping methods would only pick up the predominant serotype and that in case of co-colonisation this was always the vaccine serotype. *Points with 95 ` confidence bounds* represent data, and *lines with ribbons* represent median model estimates with 95% credible intervals. In **b** the *grey line* indicates the prior density distribution and the *bars* the posterior sample

Adaptive Markov chain Monte Carlo methods were used to fit the model to the observed data (Fig. 1) [26]. A Poisson likelihood was used for IPD, and a multinomial likelihood was used for carriage prevalence. We used a Metropolis-Hastings algorithm to create samples from the posterior parameter distributions. Prior information was used according to their availability as described earlier (Table 1 and Fig. 1).

### Vaccination scenarios

After fitting the model parameters to match the observed rates of pneumococcal carriage and disease in the KHDSS, we created multiple hypothetical PCV introduction scenarios to determine what would have happened if PCV10 had been introduced using different catch-up strategies. For this we define three alternative vaccination scenarios which assumed that administration of vaccines followed exactly the vaccine uptake that was observed in Kilifi for the respective age groups and assumed that no vaccines were administered outside the age range targeted by the scenario. These scenarios were:

1. U5 catch-up (observation and extrapolation): Vaccination according to observed vaccine coverage in KHDSS (i.e. all children under 5 years of age)
2. U2 catch-up (hypothetical): Vaccination according to observed vaccine coverage in KHDSS for all children under 2 years of age
3. U1 catch-up (hypothetical): Vaccination according to observed vaccine coverage in KHDSS for all children under 1 year of age
4. Cohort introduction (hypothetical): Vaccination according to observed vaccine coverage in KHDSS only for those children eligible for vaccination through cohort introduction

### Sensitivity analysis

We studied how competition, the duration of protection, the relative protection of infants if compared to toddlers and the vaccine efficacy against carriage and IPD within the range of their posterior distribution affected our main outcome, i.e. the number of vaccine doses needed to prevent a case of IPD (NVN) and the ratio of NVNs of the considered introduction strategies. We used a multivariable linear regression model on the centred posterior samples and reported the 95% credible interval limits of the joint distribution of the respective parameter posterior and the model coefficient as a measure of the sensitivity of the NVN ratio to the considered parameters.

We separately assessed the sensitivity of our finding to variable coverage levels in a univariate sensitivity analysis. Rather than using the observed coverage levels in Kilifi, for this we assumed that protection through routine immunisation as well as the catch-up campaign achieved either 80%, 60% or 40% coverage.

## Results

**Fig. 2:**
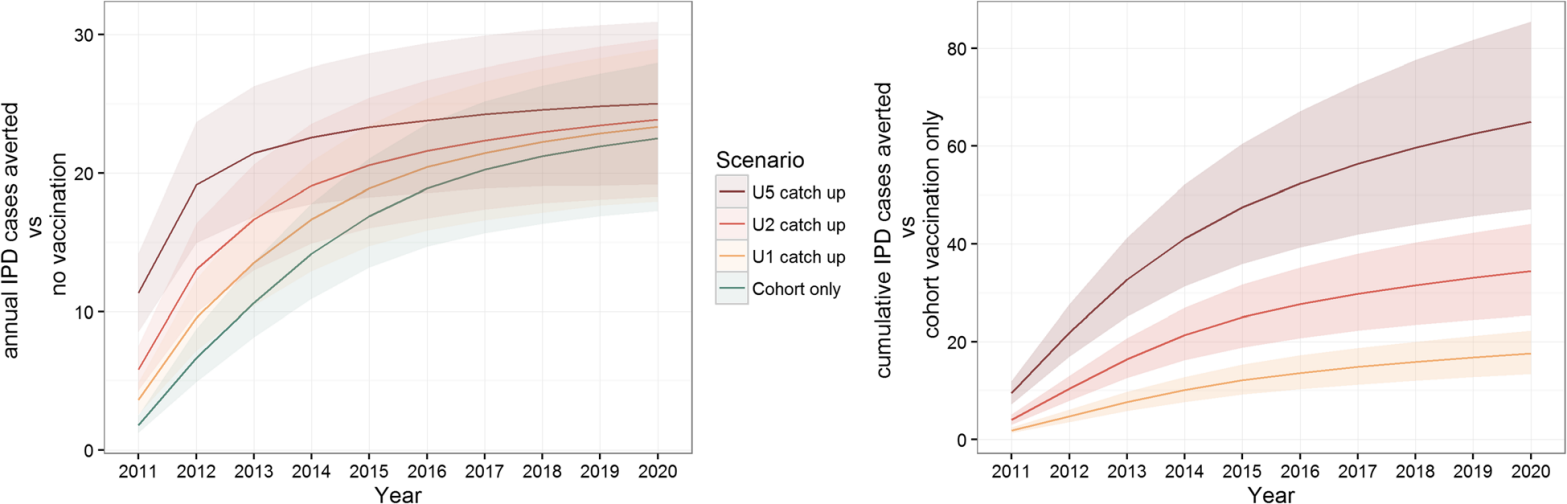
The predicted number of cases averted by PCV10 vaccination in Kilifi if introduced with a catch-up campaign in children younger than 5, 2 or 1 year old and without catch-up campaign. *Lines* represent median estimates and *ribbons* 95% credible intervals

Our model was able to reproduce Kilifi’s pre- and post-vaccination epidemiology of near VT elimination across age groups and serotype replacement with NVTs (Fig. 1). The introduction of PCV10 together with a catch-up campaign in children under 5 years old was predicted to prevent 220 (172–270) cases of IPD in Kilifi within the first 10 years after the start of the vaccination programme. Once the full direct and indirect effects (herd protection and serotype replacement) of the programme are established, the vaccination programme is predicted to avert 23 (17–28) cases of IPD annually (Fig. 2a); the majority of those among children younger than 5 years old (Fig. 1 and Additional file 1: Figure S1).

The catch-up campaign among children up to 5 years of age was estimated to accelerate direct and indirect effects of PCV. By doing so the Kilifi programme was estimated to prevent an additional 65 (48–84) cases of IPD (Fig. 2b) over 10 years in the overall population, if compared to a cohort introduction without a catch-up campaign. A catch-up programme confined to children younger than 2 years or younger than 1 year of age was estimated to prevent an additional 34 (26–43) or 18 (14–22) IPD cases, respectively, if compared to cohort introduction alone. The majority of cases averted by the catch-up campaigns would have occurred within the first 6–8 years after the start of vaccination (Fig. 2a). The age distribution of cases averted through catch-up campaigns was similar to that averted through routine immunisation (Additional file 1: Figure S1).

**Fig. 3:**
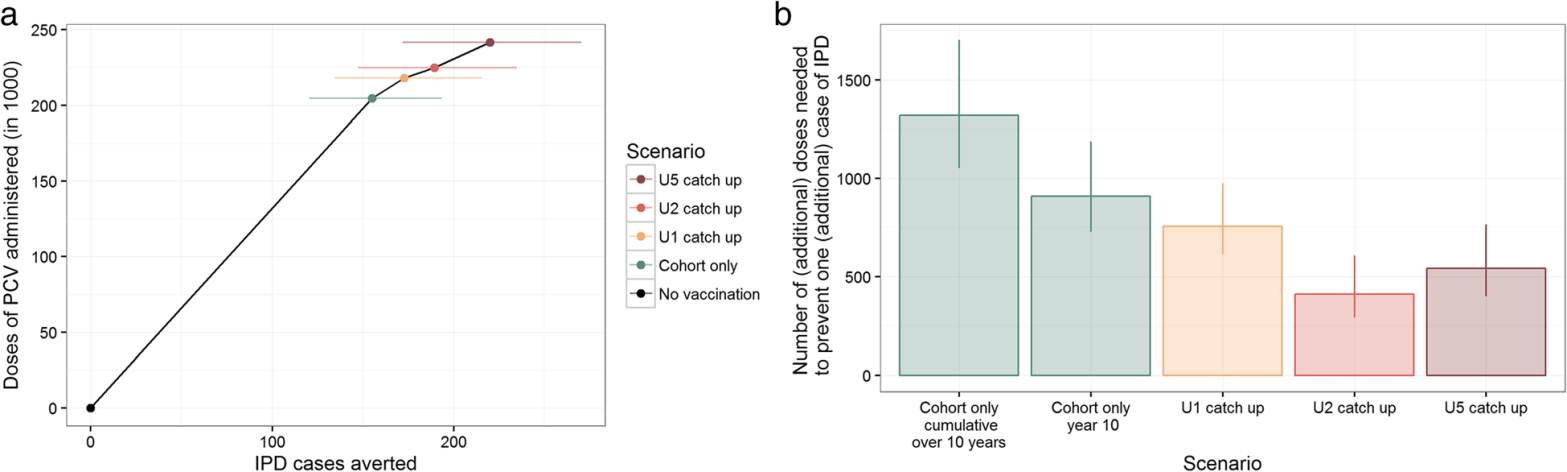
The predicted number of IPD cases averted by PCV10 vaccination in Kilifi with respect to the number of doses administered. In the dose-efficacy plane (**a**) the aggregated dose efficiency of the alternative introduction strategies within 10 years after the start of vaccination is shown. *Coloured dots and lines* represent medians and 95% credible intervals (the number of doses administered is fixed as taken from the health register). **b** shows the (incremental) number of doses needed to prevent one (additional) case of IPD. Figures for cohort vaccination alone and cohort vaccination in year 10 are presented as absolute values; the catch-up scenarios are presented as incremental values over the next smaller campaign

Within the first 10 years of the PCV infant programme in Kilifi, about 205,000 doses of vaccine were predicted to be used as part of the routine immunisation schedule. The under 5 catch-up campaign required 17,000 additional doses of vaccine (Fig. 3a). We estimated that, in the 10 years following introduction of PCV10 in Kilifi, routine vaccination without any catch-up campaigns would use 1321 (1058–1698) doses of PCV for each case of IPD averted. As herd protection gradually develops, this programme gains efficiency in the first years after introduction; that is, the annual number of cases prevented increases while the number of vaccinated individuals remains similar (Fig. 2a). By the 10th year after the start of cohort vaccination without a catch-up campaign, we estimated that routine vaccination uses 910 (732–1184) doses of PCV per IPD case averted.

The number of vaccine doses needed to prevent a case of IPD under the four scenarios is shown in Table 2. Extending catch-up PCV immunisation to children in the second year of life has the largest marginal efficiency, but any catch-up campaign is more efficient than routine birth cohort immunisation. The most efficient introduction strategy for PCV is introduction alongside an under 5 year old catch-up. All differences between schedules were found to be significant; i.e. in assessing the relative NVN, PCV introduction including a catch-up in children younger than 1, 2 or 5 years old was 4.6% (3.9–5.2), 6.2% (3.7–20.0) and 8.0% (4.5–12.9) more efficient than introduction including no catch-up or catch-up in children younger than 1 or 2 years old, respectively. Similarly, the numbers of additional vaccine doses needed to prevent an additional case of IPD via a catch-up in children younger than 1, 2 or 5 years old were 0.57 (0.54–0.61), 0.55 (0.40–0.73) and 1.32 (1.22–1.44) of those needed to prevent an additional case via cohort introduction alone, catch-up in children younger than 1 or 2 years old, respectively.

**Table 2:** The impact and efficiency of alternative introduction strategies

Introduction of PCV via	IPD averted after 10 years	Doses administered	Incremental NVN	NVN
Cohort only	155 (121–193)	204,671	1321 (1058–1698)	1321 (1058–1698)
+ U1 catch-up	173 (134–216)	218,089	757 (618–973)	1263 (1012–1623)
+ U2 catch-up	189 (147–235)	224,952	412 (296–606)	1188 (958–1527)
+ U5 catch-up	220 (172–270)	241,546	543 (403–763)	1098 (894–1405)

The number of vaccine doses needed to prevent a case of IPD (NVN) is used as a measure of efficiency. Incremental NVN refers to the additional number of doses needed to prevent one additional cases of IPD in respect to cohort introduction with the next smaller catch-up

Our results were not sensitive to variations in vaccine coverage (Additional file 1: Figure S4 and Table S1) or competition, the duration of protection, the relative protection of infants by PCV as compared to toddlers and the vaccine efficacy against carriage and IPD (Additional file 1: Figure S3).

## Discussion

In many high-income countries PCVs have been introduced with the help of catch-up campaigns to accelerate the direct and indirect protection that is offered to the community [27–29]. We used extensive data from the KHDSS, a well-studied mix of rural and urban Kenyan communities representing a typical low-income setting, to estimate the incremental effects that different catch-up campaigns are likely to have over routine vaccination and, therefore, whether PCV catch-up campaigns are an efficient use of PCV supply. We found that rapidly increasing the protection in the community via catch-up not only reduces cases of IPD by direct protection of older children but also reduces the burden of IPD in the whole childhood population by developing herd protection more rapidly. Any of the three catch-up programs considered in the analysis were estimated to use fewer vaccine doses to prevent a case of IPD than cohort introduction during the first 10 years; the catch-up schedules were more efficient than the routine cohort vaccination programme alone even after full herd effects were in place, in the 10th year of the programme. While the additional catch-up doses given to 1 year olds were estimated to provide the largest marginal efficiency, we find that cohort introduction alongside a catch-up campaign in children under 5 years old was the most efficient introduction strategy overall.

Data on the observed impact of PCV catch-up campaigns are sparse and mostly circumstantial. Catch-up campaigns of different sizes have been used for introducing PCVs into countries including the UK [30], USA [31], Israel [32], Brazil [33] and Kilifi, Kenya [11]. However, a head-to-head comparison with cohort introductions that would allow an evaluation of the additional impact of the catch-up is challenging because of the dissimilarity of the underlying population and other factors including vaccine coverage, intensity of pneumococcal transmission, differences in demographic structure and population mixing, serotype distribution and prevalence of epidemiological risk factors such as HIV infection.

As well as extending direct protection to older children who are also at high risk of pneumococcal disease, catch-up campaigns also rapidly increase the proportion of individuals in the transmitting population who are protected against VT acquisition and hence onward transmission. This indirect effect is non-linear, preventing a high number of infections for each increment in vaccine coverage when that coverage is low but suffering from a saturation effect for higher coverage levels. As a result, predictions of the optimal extent of catch-up campaigns need to account for these non-linear effects; i.e. they need to incorporate transmission dynamics.

Most of our posterior estimates that had an informative prior were similar to that prior, showing that in most instances the model is able to match the data well using the pre-specified parameter space. The notable exception was the vaccine efficacy against carriage in toddlers. While the model was unable to replicate the observed steep decrease in VT prevalence following vaccination using the mean prior estimate of 36% efficacy, the posterior suggests a mean efficacy of 55% which has been observed in other sites [23] and falls well into the range of the prior estimate.

We have restricted our analyses to catch-up campaigns that targeted age groups under 5 years of age, as those were deemed feasible both from a programmatic and a supply point of view. However, including older children may well be efficient, in particular in settings where older children contribute substantially to the transmission of pneumococci. Also, we have not considered programmatic issues associated with implementation of catch-up campaigns. Due to the immense additional burden on available staff, catch-up campaigns can disrupt routine immunisation services. Furthermore, we have studied the most efficient use of PCV10 supply but not the cost-effectiveness or affordability of catch-up campaigns for PCV10 introduction. One of the major differences in a cost-effectiveness analysis is that it takes into account the higher delivery costs of vaccine through a supplementary immunisation activity. Assuming that doses delivered as part of a PCV10 catch-up campaign were up to 75% more expensive than doses delivered through the routine EPI schedule, however, did not qualitatively change our findings on the superior efficiency of catch-up programmes.

We did not account for population growth in our model, which may impact the transmission dynamics in the post-vaccination era and hence on our findings. However, modelling work predicting the impact of PCV10 in Kilifi from pre-vaccination data has shown that accounting for population growth in Kilifi is unlikely to qualitatively change the prediction but only slightly reduces the long-term impact of vaccination on IPD [34]. As the impact of a catch-up campaign is mostly visible within a few years after vaccination, it is likely largely unaffected by long-term changes in demographics. Hence, accounting for population growth is likely to further favour the use of catch-up campaigns for introduction of PCV. Other models have taken into account more of the diversity of pneumococcal serotypes by either modelling them individually or by using finer groupings [19, 34–36]. Despite the considerable heterogeneity of serotypes in regard to their ecology within both our VT and NVT group, our model captures the post-vaccination dynamics well. The impact of catch-up campaigns largely concerns the acceleration of long-term impact of the programme, and hence nuances in the dynamics of specific serotypes are unlikely to qualitatively change our findings. We have not taken into account potential underreporting of IPD episodes. However, extra attention has been paid to that during the study period; the same procedures were followed at Kilifi hospital so that ascertainment has not changed, and hence any such bias in our estimates for relative impact of PCV introduction strategies should be minimal. Further, our primary objective was to estimate the relative impact of PCV10 introduction with and without catch-up campaigns. Hence, we chose not to incorporate factors that influence the absolute impact of pneumococcal vaccination but that should make little difference to the impact of catch-up campaigns relative to cohort introduction, such as impact on otitis media, pneumonia or mortality, secular trends, antibiotic consumption, comorbidities or cost. We fit age-group- and serotype-group-specific pathogenicity and assumed that those would stay unchanged during the study period. While vaccination may have led to a disproportionate emergence of some serotypes and hence changes in serotype-group pathogenicity, Nurhonen and Auranen have shown that the assumption of proportional expansion and hence unchanged pathogenicity generally holds [37], and our model fits well to both pre- and post-vaccination IPD and carriage without the need for allowing a change in pathogenicity.

The generalisability of our results beyond KHDSS is dependent on a number of factors. We show in a sensitivity analysis the robustness of our findings to vaccine coverage, vaccine efficacy against the carriage and IPD, the ratio of toddler to infant protection, the duration of vaccine-induced protection and the between-serotype group competition (see Additional file 1: Figure S3 and Figure S4). However, other factors that could not be systematically assessed in this analysis include transmission intensity and serotype distribution. In settings with higher transmission intensity, birth cohort PCV introduction likely takes longer to establish full herd effects. As a result, catch-up campaigns that accelerate the build-up of herd protection have the potential to prevent more IPD cases and hence be an even more efficient strategy for PCV use. Furthermore, we did not account for potential cross-reactivity of PCV10 against serotypes 6A and 19A, which has been reported previously [38, 39]. However, both 6A and 19A carriage prevalence increased in the post-vaccination era in Kilifi [11].

We assumed that two PCV10 doses in infancy, given as part of the routine EPI schedule, are similarly efficacious at preventing VT carriage and disease as a single catch-up dose in toddlers and young children. Fitting to the data from Kilifi, our model did not reject this hypothesis. While our results are robust to factors including these differences in relative protection in infants and toddlers and variable vaccine coverage (proportion of protected infants and toddlers), the number of doses that are administered to establish protection could have a larger impact. Twelve months after the introduction of PCV10 in Kilifi, 76% of infants eligible for three doses of PCV10 aged less than 1 year had received at least two doses of PCV10, and 62% of children 1–4 years old had received at least one dose. We have chosen the dosing of catch-up campaigns to align with what was rolled out in Kilifi; however, other dosing regimens have been used [40], notably in south Africa with two doses in infancy followed by a booster dose at 9 months of age [41]. In our analysis we assume for simplicity that all children receive the exact number of doses that in this analysis was deemed sufficient to induce protection. Drop-out rates in Kilifi are relatively low; e.g. more than 97% of infants who received one dose go on to receive a second dose before 1 year of age, but including drop-outs in the analysis would further decrease the efficiency of the cohort introduction in comparison to the catch-up campaigns. To define protection in our model, we used two doses in infancy and one dose for catch-up campaigns as a protective schedule but assumed that vaccinated children would eventually receive three doses as part of the routine schedule or alternatively two doses or one dose if part of the catch-up campaign in <1 year old or older children, respectively. Assuming instead that the children protected through routine immunisation and catch-up campaigns had received two doses and one dose, respectively, did not qualitatively change the results.

## Conclusions

Pneumococcal conjugate vaccines (PCVs) are among the most expensive vaccines currently available and make up more than 30% of the annual budget of Gavi. Proposed ways to use PCVs more efficiently include a potential reduction in the number of infant doses if herd effects have been established [42] or a dilution of the current formulation. We show here that catch-up campaigns present an important, readily available tool which can increase the efficiency of the PCV’s impact on disease at introduction. For countries yet to introduce, or potentially also for countries with lagging coverage, strategies that include catch-up campaigns warrant serious consideration.

## Additional file

Additional file 1: Supplemental materials. (DOCX 1719 kb).
