# Supplementary material for: Assessing the efficiency of catch-up campaigns for the introduction of pneumococcal conjugate vaccine: a modelling study based on data from PCV10 introduction in Kilifi, Kenya
Source: BMC Med. 2017 Jun 7;15:113. doi: 10.1186/s12916-017-0882-9 (PMC5463405; doi:10.1186/s12916-017-0882-9)

**Supplement to

“Assessing the efficiency of catch-up campaigns for introduction of pneumococcal conjugate vaccine; a modelling study based on data from Kilifi, Kenya”**

**Model equations**


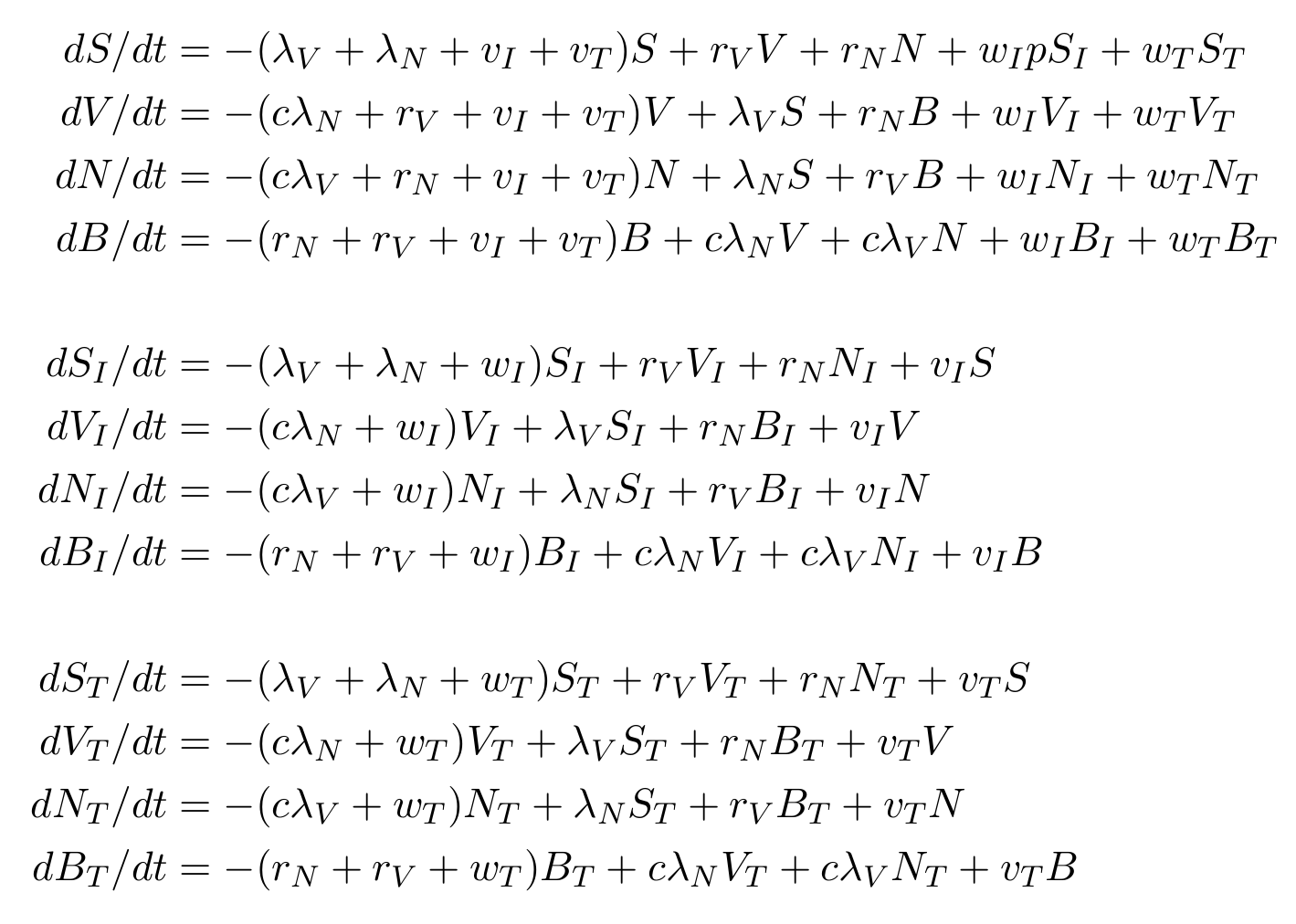


Age and time dependencies were omitted for clarity. S, V, N, B indicate Susceptible, Vaccine type carriers, Non-vaccine type carriers, and carries with both vaccine and non-vaccine types and subscripts represent the status of vaccine protection: I and T represent infant and toddlers protection. An overview of the parameters and their meaning is given below.


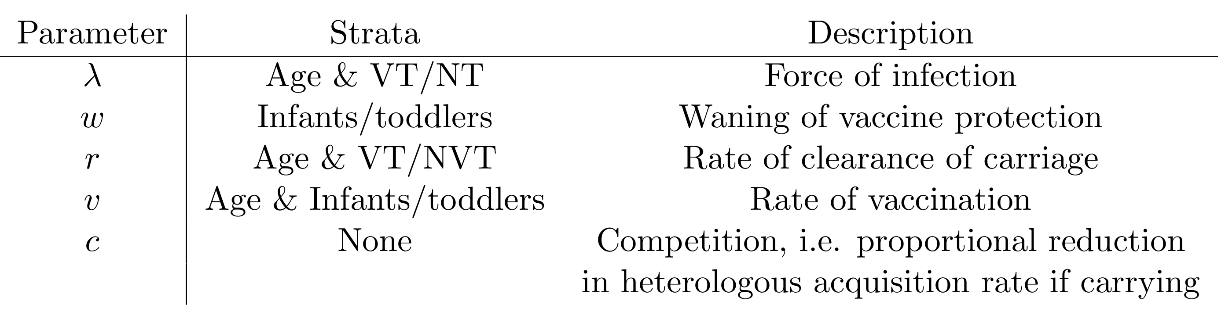


**Posteriors for all parameters**

| **Parameter** | **<1yrs** | **1-5yrs** | **6-14yrs** | **15yrs+** |
| --- | --- | --- | --- | --- |
| **Probability of infection with VT if contacted ( in 10^-3^)** | 3 (2-4) | 6 (5-8) | 3 (2-4) | 1 (1-2) |
| **Probability of infection with NVT if contacted (in 10^-3^)** | 4 (3-5) | 7 (6-8) | 4 (3-4) | 2 (2-2) |
| **Case to carrier ration of VT (in 10^-5^)** | 68 (42-108) | 24 (18-32) | 4 (2-6) | 2(1-3) |
| **Case to carrier ration of NVT (in 10^-5^)** | 15 (10-22) | 2 (1-3) | 0 (0-1) | 0 (0-0) |
| **Reduction in heterologous acquisition if carrying** | 0.19 (0.08-0.43) | | | |
| **Vaccine efficacy against carriage** | 0.56 (0.42-0.72) | | | |
| **Vaccine efficacy against disease if carrying** | 0.70 (0.25-0.97) | | | |
| **Relative protection of infants** | 0.98 (0.79-1.16) | | | |
| **Duration of protection (yrs)** | 5.8 (2.1-11.2) | | | |

Figure S1: Model fit to data and age distribution of averted cases. Top panel: Model fit to carriage prevalence and IPD incidence. We assumed that serotyping methods would only pick up the predominant serotype and that such was always the vaccine serotype. Points with 95% confidence bounds represents data and lines with ribbons represent median model estimates with 95% credible intervals. Lower panel: Incremental number of IPD cases averted stratified by age group. The age distribution of additional cases averted through routine immunization, U1 catch-up, U2 catch-up, U5 catch-up vs no vaccination, routine immunization, U1 catch-up, U2 catch-up respectively.

**
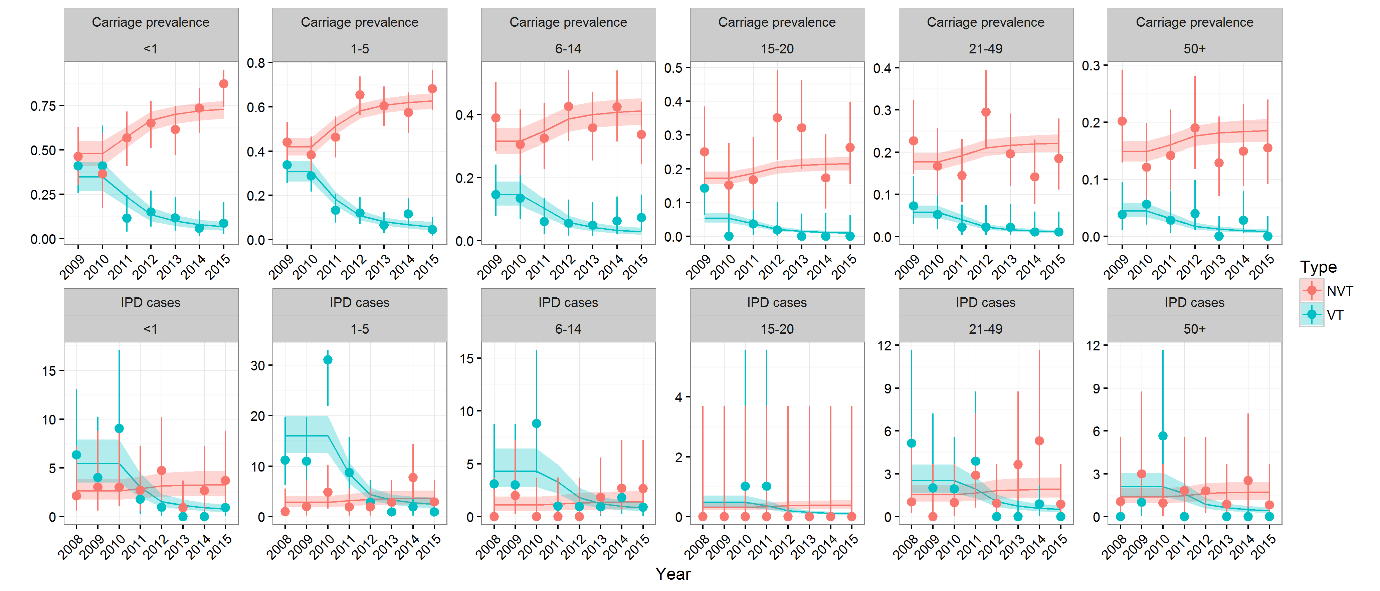
**

**
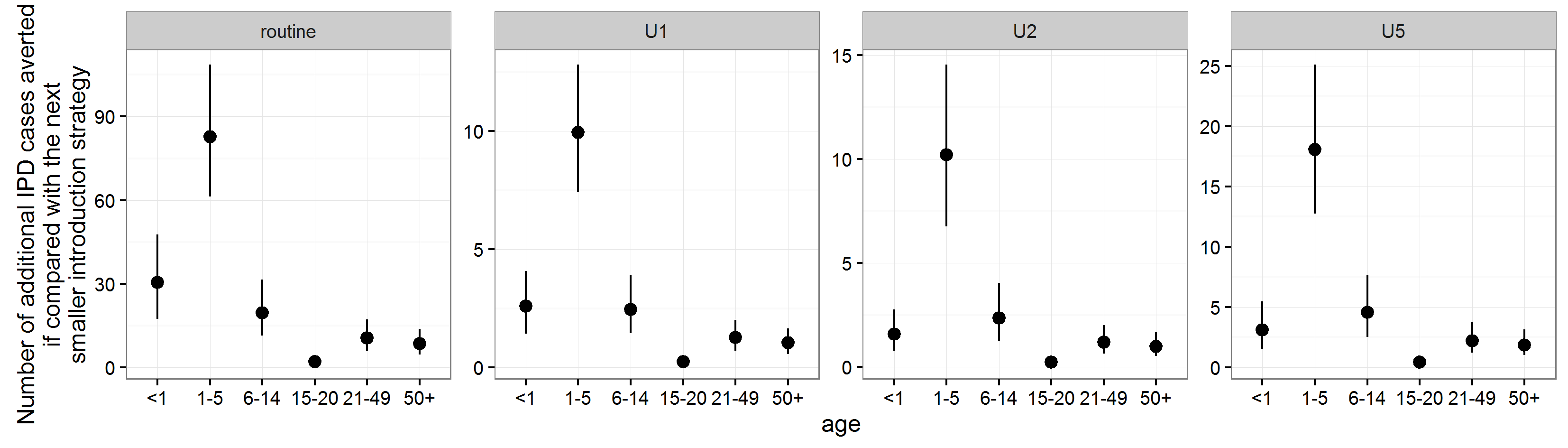
**

Figure S2: Observed vaccine coverage in age and time in Kilifi in Infants (upper panel) and older children (lower panel). Beyond 100 weeks after PCV introduction coverage trends were extrapolated based on the observed coverage in week 100.


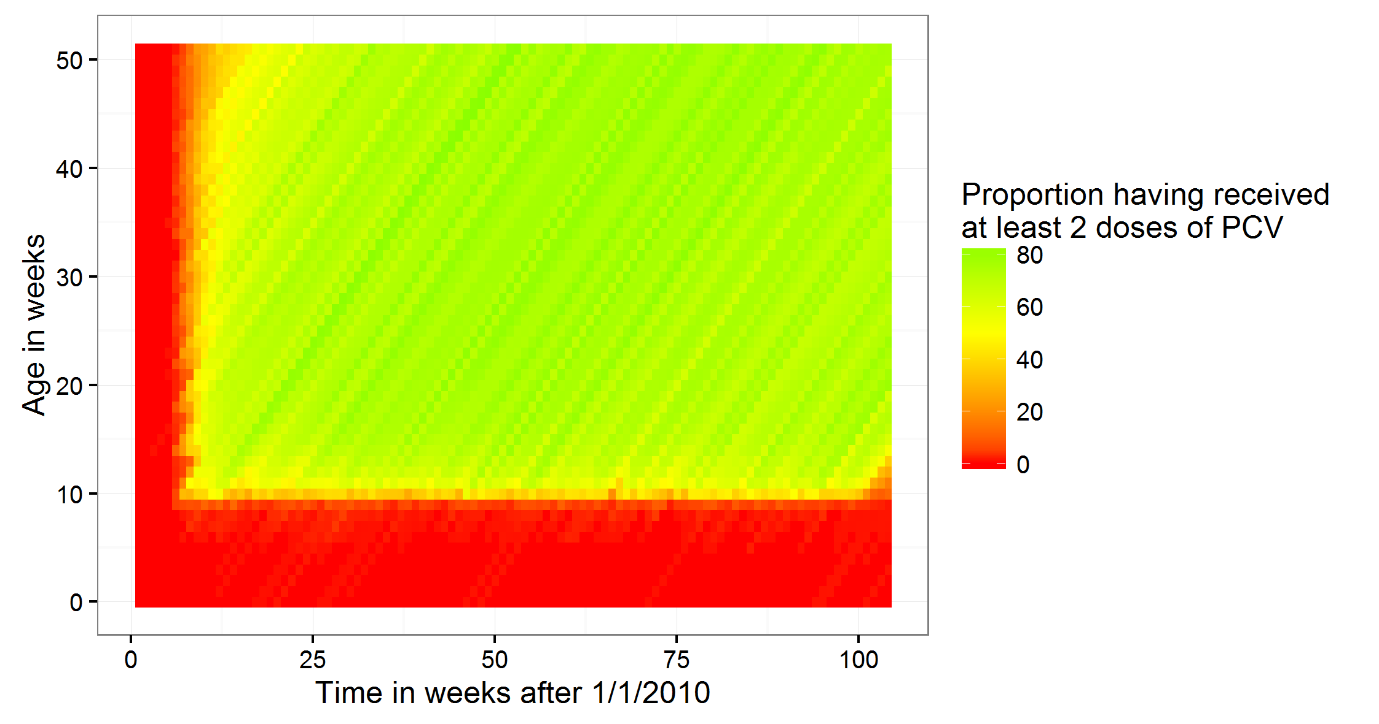

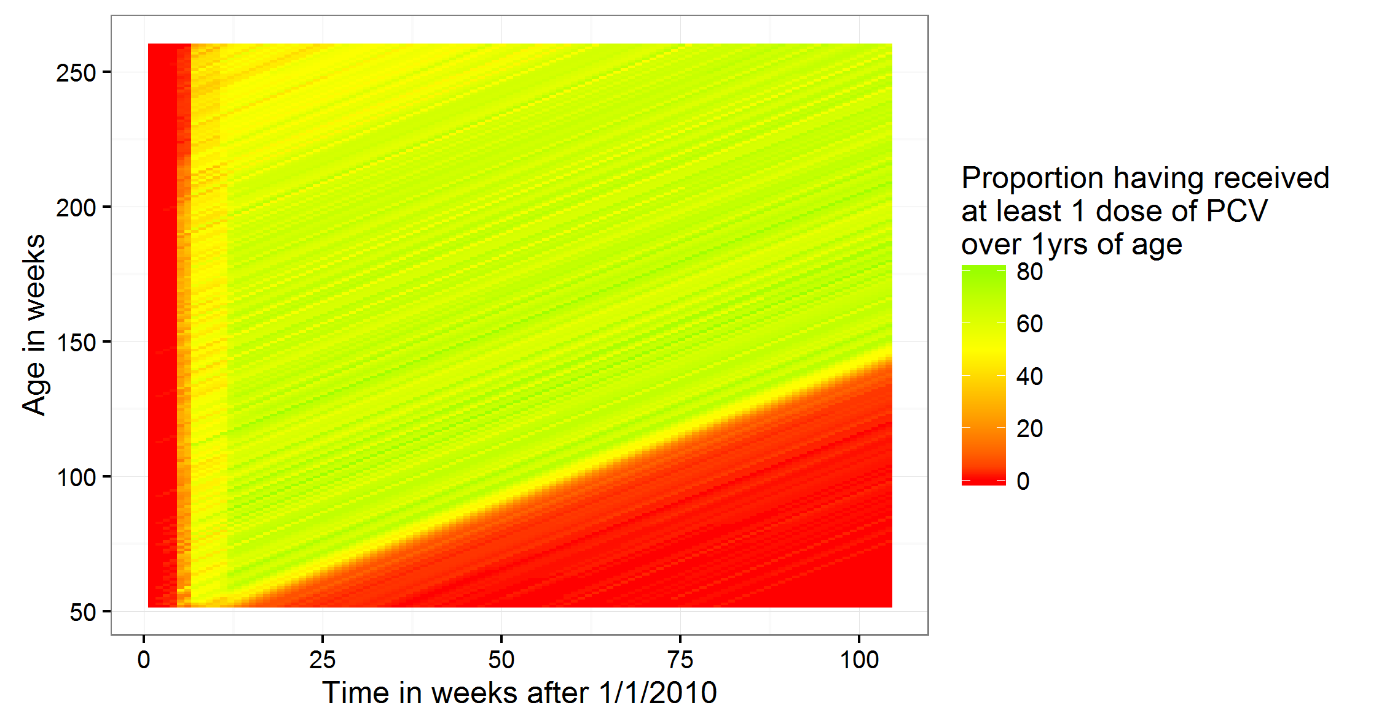


**Sensitivity analysis**

Table S1: The sensitivity of the efficiency of alternative introduction strategies to vaccine coverage. The number of vaccine doses needed to prevent a case of IPD (NVN) is used as a measure of efficiency.

| Introduction of PCV via | NVN Kilifi coverage | NVN 40% coverage | NVN 60% coverage | NVN 80% coverage |
| --- | --- | --- | --- | --- |
| Cohort only | 1321 (1058 to 1698) | 1302 (1036 to 1664) | 1346 (1079 to 1716) | 1387 (1118 to 1764) |
| + U1 catch-up | 1263 (1012 to 1623) | 1238 (977 to 1581) | 1283 (1027 to 1644) | 1326 (1070 to 1685) |
| + U2 catch-up | 1188 (958 to 1527) | 1159 (918 to 1483) | 1202 (967 to 1538) | 1251 (1024 to 1592) |
| + U5 catch-up | 1098 (894 to 1405) | 1035 (820 to 1325) | 1085 (867 to 1382) | 1162 (962 to 1457) |

Figure S3: Sensitivity of the number of vaccine doses needed to prevent one case of IPD (NVN) ratios between doses given as part of alternative introduction strategies (incremental). The dashed line shows the median ratio of NVN. The bars show the 95% credibility range of the joint distribution of the parameter posteriors and the linear regression model’s estimated coefficient.


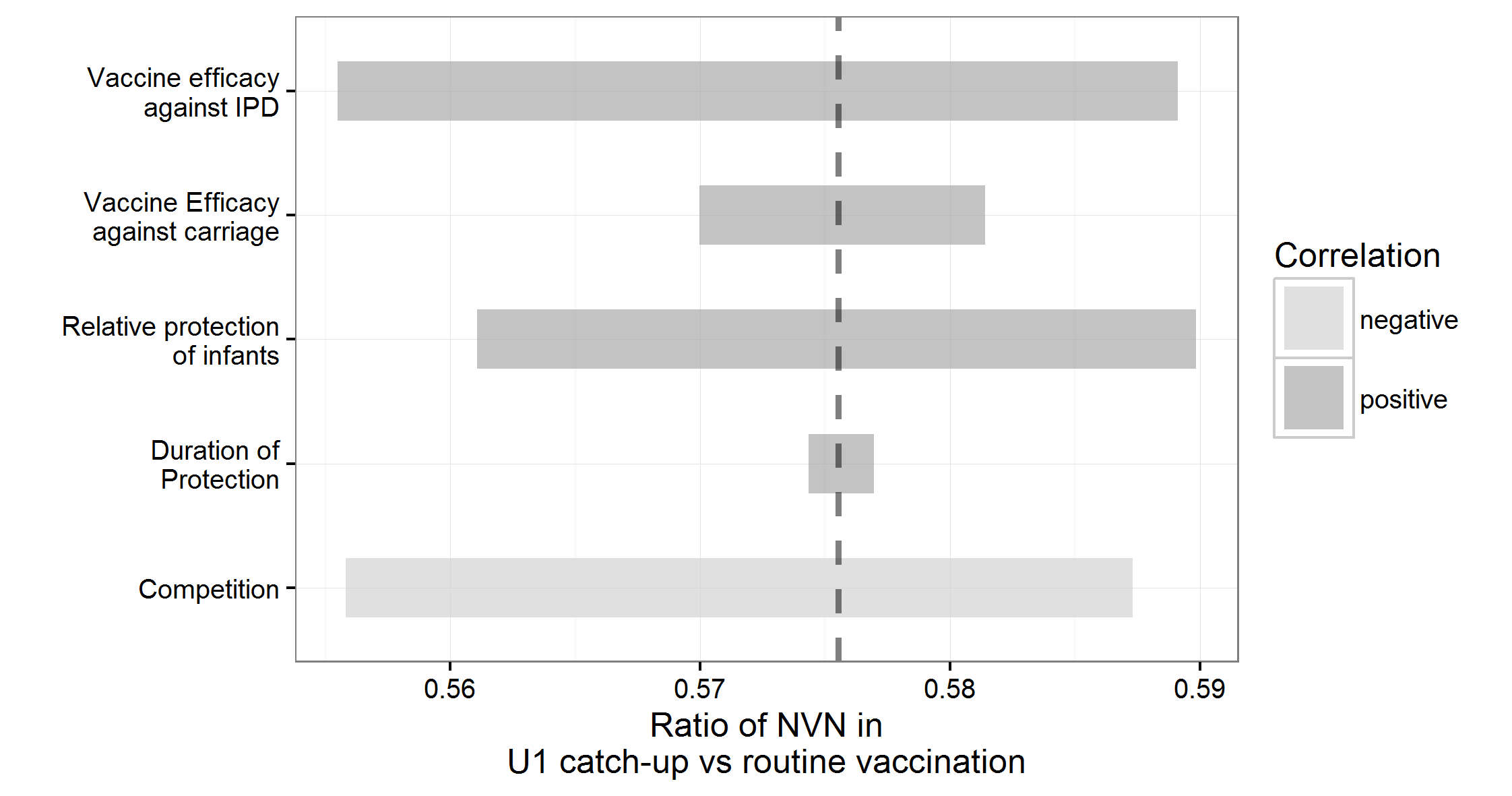


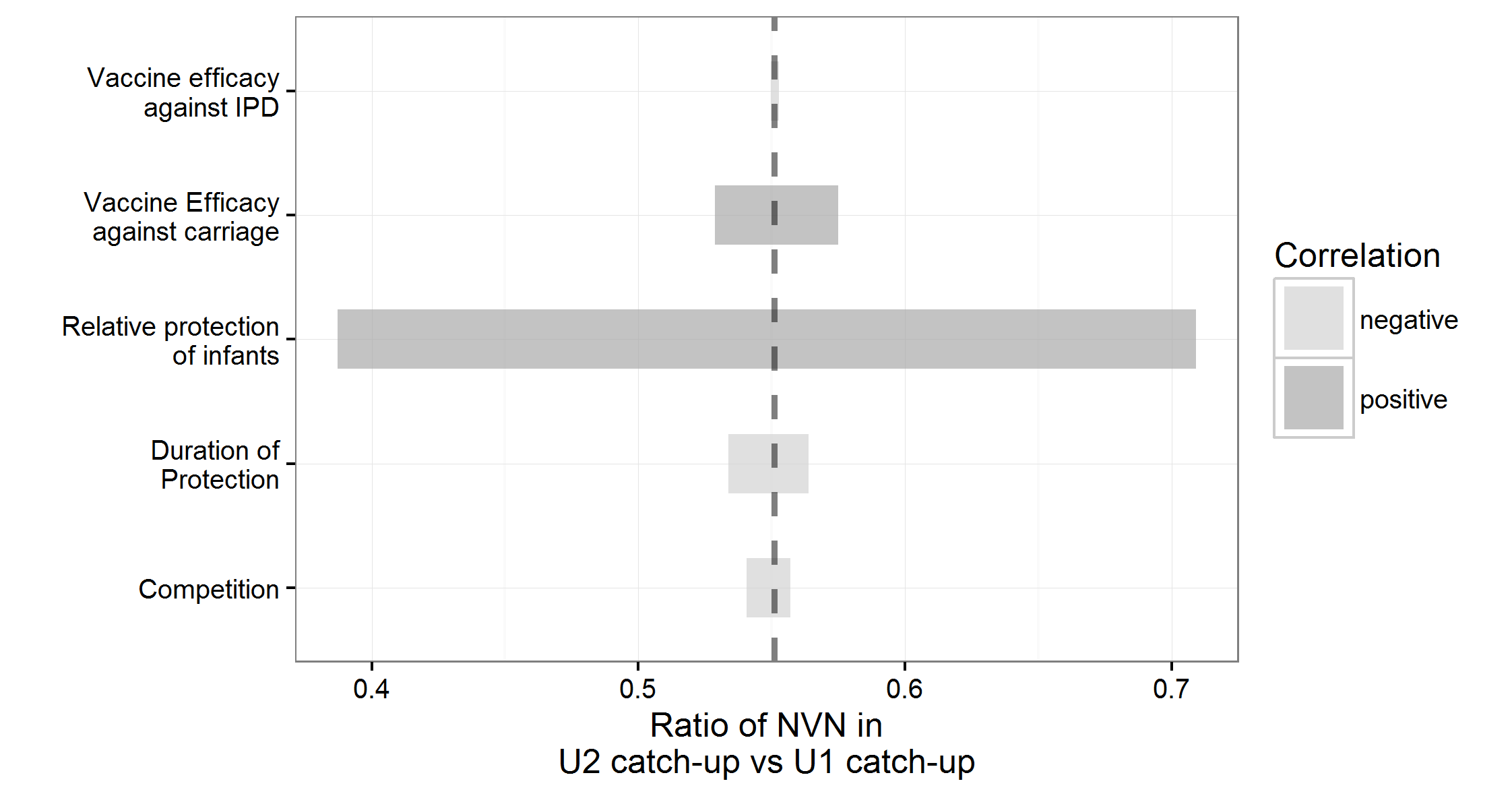

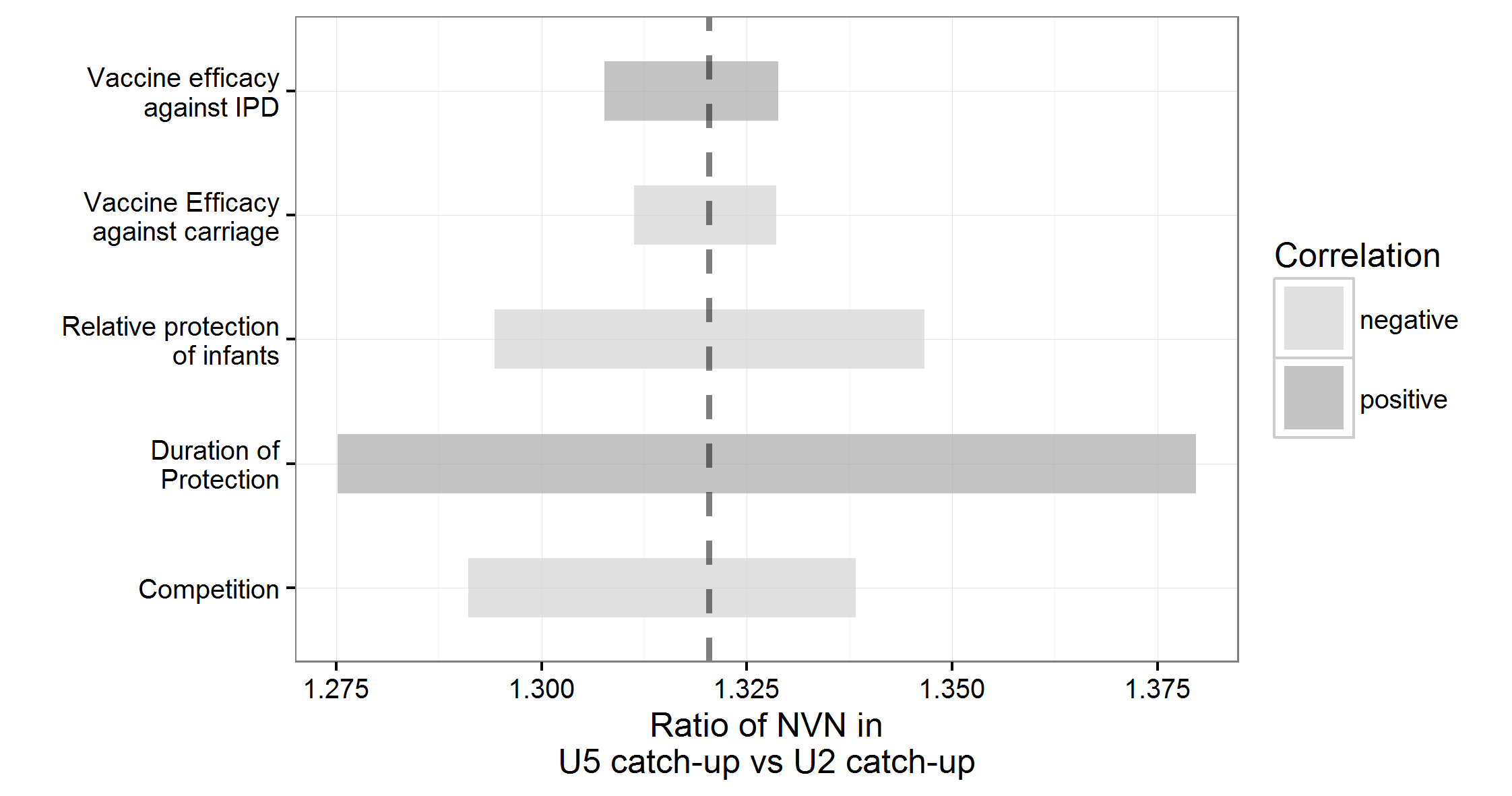


Figure S4: The predicted number of IPD cases averted by PCV10 vaccination in Kilifi in respect to the number of doses administered for the sensitivity scenarios of 40%, 60% and 80% coverage (top to bottom). In the dose-efficacy plane (upper panel) the aggregated dose-efficiency of the alternative introduction strategies within 10 years after the start of vaccination is shown. Coloured dots and lines represent medians and 95% credible intervals (the number of doses administered is fixed as taken from the health register). In the lower panel the (incremental) number of doses needed to prevent one (additional) case of IPD. Figures for cohort vaccination alone and cohort vaccination in year 10 are presented as absolute values, the catch-up scenarios are presented as incremental values over the next smaller campaign.


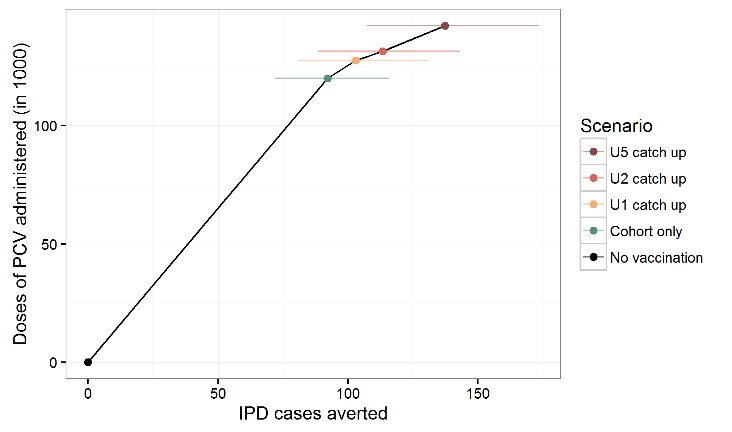


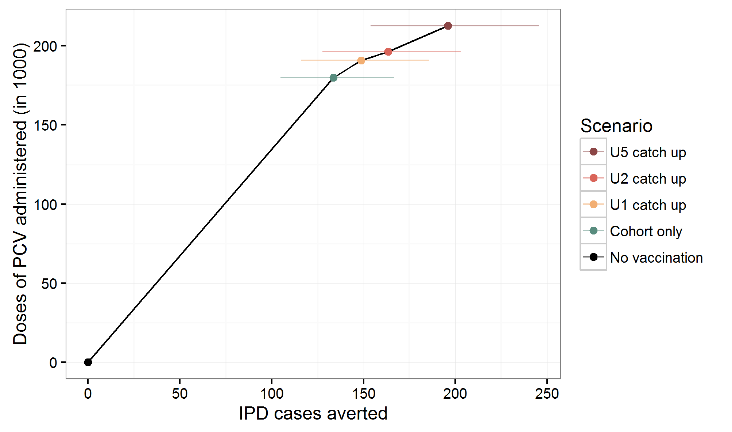


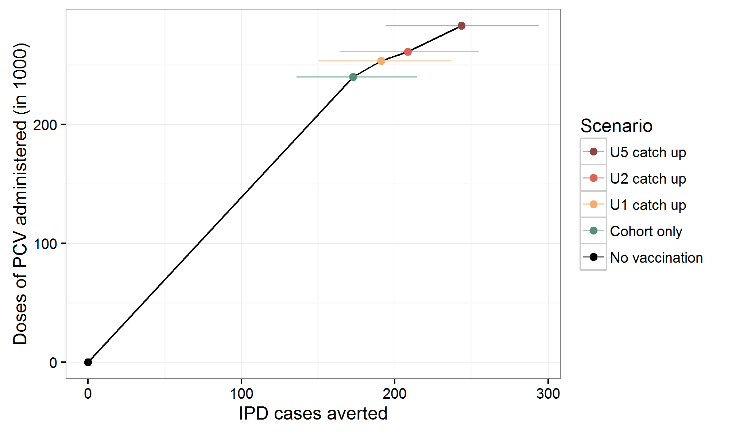

Supplement: Additional file 1: — Supplemental materials. (DOCX 1719 kb). [file 12916_2017_882_MOESM1_ESM.docx]
